# Supplementary material for: Knockdown resistance allele L1014F introduced by CRISPR/Cas9 is not associated with altered vector competence of Anopheles gambiae for o’nyong nyong virus
Source: PLoS One. 2023 Aug 10;18(8):e0288994. doi: 10.1371/journal.pone.0288994 (PMC10414658; doi:10.1371/journal.pone.0288994)
Supplement: S1 File — (DOCX) [file pone.0288994.s001.docx]

## Cytopathic effects assay sensitivity analysis

Cytopathic effects (CPE) assays provide a high throughput, non-quantitative method for determining whether virus is present in mosquito body parts and saliva. This method is used to screen out virus-negative samples to avoid performing unnecessary plaque assays. The sensitivity and limit of detection (LOD) of this assay was determined for ONNV UgMp30 virus in Vero cells.

Method

A dilution series was prepared from stocks of ONNV and inoculated onto cells from tandem CPE and plaque assay analysis. CPE and plaque assays were performed as described in the main methods section of the manuscript. Duplicates for each dilution were performed on plaque assay. Each dilution had 7 replicates on the CPE assay. Plaque assays were incubated for 48h prior to fixing, staining and counting of plaques. CPE assays were incubated for 72h and scored by microscopy.

Results

The sensitivity analysis using paired CPE and plaque assays show that CPE assays down to very low titres. Cell death and other cytopathic effects (e.g. cell rounding, detachment, and sloughing) were observed in 100% of CPE replicates down to a titre of 1.9x10^3^ PFU/mL which equates to approximately 4-5 PFU per 25µL inoculum added to the assay. Even at titres as low as 25 PFU/mL (<1 PFU on average per 25µL inoculum), at least 1 CPE replicate remained positive (**Supplementary Table 1**).

| **Supplementary Table 1** – Cytopathic Effects Assay Sensitivity Analysis | | | | | | | | | | | |
| --- | --- | --- | --- | --- | --- | --- | --- | --- | --- | --- | --- |
| **Dilution number** | **1** | **2** | **3** | **4** | **5** | **6** | **7** | **8** | **9** | **10** | **11** |
| **Mean number of plaques** | 28.5 | 18 | 25 | 17 | 27 | 21 | 19 | 18 | 5.5 | 2.5 | 0 |
| **Titre (PFU/mL)** | 2.9 x10^5^ | 1.8 x10^5^ | 2.5 x10^4^ | 1.7 x10^4^ | 2.7 x10^3^ | 2.1 x10^3^ | 1.9 x10^3^ | 1.8 x10^2^ | 55 | 25 | 8.3* |
| **Percentage of CPE replicates positive (%)** | 100 | 100 | 100 | 100 | 100 | 100 | 100 | 71.4 | 42.9 | 28.6 | 0 |
| **PFU in 100µL inoculum for plaque assay** | 2.9 x10^4^ | 1.8 x10^4^ | 2.5 x10^3^ | 1.7 x10^3^ | 270 | 210 | 19 | 18 | 5.5 | 2.5 | 0.8 |
| **PFU in 25µL inoculum for CPE assay** | 7.1 x10^3^ | 4.5 x10^3^ | 625 | 425 | 67.5 | 52.5 | 4.75 | 4.5 | 1.4 | 0.6 | 0.2 |

*Lowest dilution was negative by plaque assay. Titre is inferred from dilution series.

Discussion

These results show that CPE assays are a sensitive method for screening mosquito samples for ONNV. At least 1 CPE replicate remained positive down to <1 PFU on average per 25µL inoculum added to the assay, meaning it is likely that the CPE assay will be positive if a single PFU is added in the inoculum. In order to avoid misidentifying samples as virus negative, the presence of a single positive CPE replicate should prompt further analysis by plaque assay. As such, it seems the CPE assay used here is a highly sensitive screening method.
